# An Overview of Bioinformatics Tools for DNA Meta-Barcoding Analysis of Microbial Communities of Bioaerosols: Digest for Microbiologists

**DOI:** 10.3390/life10090185

**Published:** 2020-09-08

**Authors:** Hamza Mbareche, Nathan Dumont-Leblond, Guillaume J. Bilodeau, Caroline Duchaine

**Affiliations:** 1 Sunnybrook Research Institute, Toronto, ON M4N 3M5, Canada; 2 Department of Laboratory Medicine and Pathobiology, University of Toronto, Toronto, ON M5S 1A1, Canada; 3 Centre de Recherche de l’Institut Universitaire de Cardiologie et de Pneumologie de Québec, Quebec City, QC G1V 4G5, Canada; nathan.dumont-leblond@criucpq.ulaval.ca; 4 Département de Biochimie, de Microbiologie et de Bio-informatique, Faculté des Sciences et de Génie, Université Laval, Quebec City, QC G1V 0A6, Canada; 5 Pathogen Identification Research Lab, Canadian Food Inspection Agency (CFIA), Ottawa, ON K2J 1G3, Canada; guillaume.bilodeau@canada.ca

**Keywords:** bioaerosols, bioinformatics, microbial ecology

## Abstract

High-throughput DNA sequencing (HTS) has changed our understanding of the microbial composition present in a wide range of environments. Applying HTS methods to air samples from different environments allows the identification and quantification (relative abundance) of the microorganisms present and gives a better understanding of human exposure to indoor and outdoor bioaerosols. To make full use of the avalanche of information made available by these sequences, repeated measurements must be taken, community composition described, error estimates made, correlations of microbiota with covariates (variables) must be examined, and increasingly sophisticated statistical tests must be conducted, all by using bioinformatics tools. Knowing which analysis to conduct and which tools to apply remains confusing for bioaerosol scientists, as a litany of tools and data resources are now available for characterizing microbial communities. The goal of this review paper is to offer a guided tour through the bioinformatics tools that are useful in studying the microbial ecology of bioaerosols. This work explains microbial ecology features like alpha and beta diversity, multivariate analyses, differential abundances, taxonomic analyses, visualization tools and statistical tests using bioinformatics tools for bioaerosol scientists new to the field. It illustrates and promotes the use of selected bioinformatic tools in the study of bioaerosols and serves as a good source for learning the “dos and don’ts” involved in conducting a precise microbial ecology study.

## 1. Introduction

The development of next-generation sequencing (NGS) platforms for DNA samples has grown exponentially in recent years [1,2,3]. This burst in high-throughput sequencing (HTS) has revolutionized our understanding of the microbial composition of a wide range of environments [4,5,6,7,8,9]. More specifically, amplicon-based sequencing is the most commonly used method for characterizing microbial diversity [10,11,12,13]. This method includes the use of a taxonomically informative genomic marker that is common to all microorganisms of interest and that is targeted by an amplification step prior to sequencing. For bacteria and archaea, amplicon-based sequencing studies target the gene that codes for the small 16S ribosomal subunit [14]. For fungi, the gene that codes for the Internal Transcribed Spacer (ITS) is considered the universal maker for the study of fungal diversity by molecular approaches [15]. The sequenced amplicons are characterized using bioinformatics tools to determine which microbes are present in a sample and at what relative abundance. Comparing the targeted sequences across samples gives insight into how microbial diversity associates with and scales across environmental conditions.

HTS approaches have been used to characterize the microbial composition of various environments, from soil, water, and the rhizosphere to the human gut [16,17,18,19]. In 2010, Peccia and his collaborators [20] highlighted the importance of incorporating DNA sequencing methods into the study of aerosol science. In fact, molecular methods have made it possible to characterize new archaeal diversity in bioaerosols, which would’ve been impossible with culture-dependent methods [21]. This opened the door to understanding strictly anaerobic archaea. Applying HTS methods to air samples from different environments allows the identification and quantification (relative abundance) of the microorganisms present and gives a better understanding of human exposure to indoor and outdoor bioaerosols. Using HTS approaches offers a thorough picture of the microbial content of aerosols and leads to millions of sequences generated from that single sample [22,23,24,25,26,27]. In order to make full use of the information made available by these sequences, repeated measurements must be taken, community composition described, error estimates made, correlations of microbiota with covariates (variables) must be examined, and increasingly sophisticated statistical tests must be conducted, all by using bioinformatics tools [28].

Bioinformatics is not new to science, as it was first mentioned back in 1970 in a conversation between Dutch scientist Paulien Hogeweg and her colleague Ben Hesper to describe their work on the study of informatic processes in biotic systems [29]. Consistent with the rise in NGS, the past few years represent a surge in bioinformatics tool development for analyzing the large amounts of data generated by amplicon-based sequencing approaches [30,31,32,33,34,35]. Bioinformatics can be divided into computational biology, which uses algorithms to build mathematical models to solve biological problems using a computational method, and analytical bioinformatics, which uses bioinformatics tools to analyze biological data [36]. This definition of bioinformatics inspired conversations about the status of bioinformaticians. Vincent and Charette tried to answer the question “Who qualifies as a bioinformatician?” by suggesting that the status should be reserved for experts who develop bioinformatics algorithms and tools (software) and for those who design architectural models to maintain databases [37]. This definition did not elicit unanimity amongst the scientists who do not develop algorithms, but who use bioinformatics tools on a daily basis to analyze data, generate results and solve problems [38]. While this distinction is important as it allows universities, human resources and governments to accurately recognize and certify students, employees and others as bioinformatics experts, it is important to remember that using computers to understand biological concepts is as important and necessary as using any other laboratory tool/equipment. Because microbiology is entering a new era, bioaerosol scientists, among others, should not fear using bioinformatics tools to conduct microbial community studies.

Knowing which analysis to conduct and which tools to apply remains confusing for bioaerosol scientists, as a litany of tools and data resources are now available for characterizing microbial communities. The goal of this review paper is to offer a guided tour through the bioinformatics tools that are useful in studying the microbial ecology of bioaerosols. This paper does not focus on sequence data processing (quality filtering, Operational Taxonomic Unit clustering, etc.) as this information is described in previously published work [25,26] and there is ample literature available on bioinformatics pipelines for processing sequences [30,32,39,40,41]. This work explains microbial ecology features like alpha and beta diversity, multivariate analyses, differential abundances, taxonomic analyses, visualization tools and statistical tests using bioinformatics tools for bioaerosol scientists new to the field.

## 2. Methods and Software

The methodological bioinformatics approaches proposed in this manuscript for studying the microbial ecology of bioaerosols rely on the use of widely adopted QIIME pipelines, Mothur software [30,32] and R packages; particularly, the vegan [42], phyloseq [43], DADA2 [44], and RAM packages (https://rdrr.io/cran/RAM/man/RAM-package.html). All of the analyses proposed in this manuscript can be done using these software programs and R packages. Detailed documentation about their usage is available online. Additionally, Bioconductor is an open-source software package for bioinformatics that offers different features, courses and training on the usage of R for sequencing data associated with microbial ecology (https://www.bioconductor.org/).

**Figure 1:**
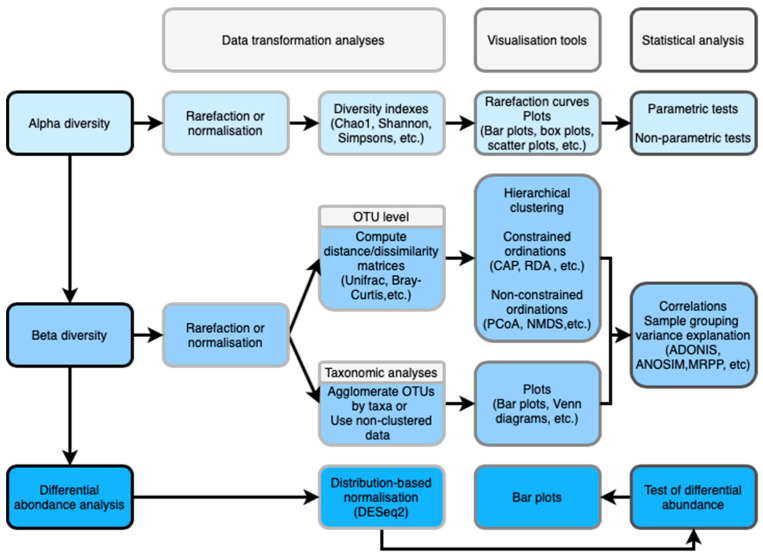
Quick overview of the microbial ecology analyses using bioinformatics tools. The figure shows the succession of analyses from alpha diversity to differential abundance and the three stages of analysis: data transformation, visualization tools, statistical analysis, of each step.

Before starting the diversity analyses, users are recommended to build a metadata mapping file. The mapping file is a tabulated text file (it can be constructed using excel or LibreOffice) that contains all of the information about the samples necessary to perform the data analysis. In general, the mapping file should contain the name of each sample, the barcode sequence used for each sample, the linker/primer sequence used to amplify the sample, and a description column. It is important to include in the mapping file any metadata related to the samples (e.g., age, gender, temperature, season, pH, etc.) and any additional information relating to specific samples that may influence the microbial content of the samples (e.g., type of samplers used). QIIME offer a guideline on how to build a metadata mapping file: http://qiime.org/scripts/validate_mapping_file.html. Figure 1 is a quick overview showing the succession of all the major steps of the microbial ecology analyses using bioinformatics tools that will be discussed in this work. Each step is divided into three stages: data transformation, visualisation and statistical analysis.

### 2.1. Controls and Bio-informatic Management of Controls

It has been reported numerous times that NGS is prone to the incorporation of contaminants, both bacterial and fungal, and that they can have a significant impact on the conclusions of studies, even more so when looking at low-microbial-biomass samples, such as aerosols samples [12,45,46,47]. These contaminants can originate from a variety of sources, including the different reagents used in the extraction protocols and even from cross contamination between samples [48]. The NGS platforms can also erroneously label nucleotides, which can lead to the misidentification of microbes [49]. Technical biases, such as preferential amplification by the primers used to prepare sequencing libraries and polymerase errors, have also been widely described [50].

Incorporating positives controls, such as a mock community, and negatives controls (such as field blanks) in a study design is now a well-spread practice in order to observe the possible biases induced by contaminants, library preparation and sequencing itself and attempt to compensate for them.

### 2.2. Mock Microbial Communities

Comparing taxonomic information of bioaerosol samples to a mock community sample can help determine technical biases linked to sequencing approaches. A mock community is a consortium of microorganisms of known composition and structure. It can be a whole-cell or DNA community, in which either the complete microorganisms or only their genomes are present. The first type can allow comparisons of extraction protocols efficiencies, while the DNA mock communities give a better insight at the library preparation, sequencing and bio-informatic analysis steps [12]. The sequencing results of these known samples can be compared with the expected data in order to observe and quantify the possible bias introduced by the method on the samples. Then, the relative abundance of the different taxa identified in the actual samples can be adjusted to take into account this observed bias. Those types of modification must be made cautiously, as they can have a major impact of the final results. For example, the latter analysis is achieved by simply comparing the relative abundance of the expected data (e.g., *Streptococcus* 20%; *Pseudomonas* 20%; *Staphylococcus* 20%; etc.) to the sequencing results after library preparation (e.g. *Streptococcus* 15%; *Pseudomonas* 22%; *Staphylococcus* 10%; etc.). Then, the relative abundance of the taxa in the samples could be readjusted by taking into account the rise or the drop of the percentage of relative abundance.

The use of mock bacterial communities is more and more frequent in the literature and they are commercially available [51]. On the other hand, mock fungal communities are not as readily available as their bacterial counterparts. Although there have been recent attempts at creating one [52], the lack of accessibility seems to refrain its implementation. Additional work must be deployed in order to create standardized communities and procedures that can become the gold standard for microbial ecology studies. In the meantime, creating your own custom-made community might be good way to get better insight of the possible biases of your methodology. Like for bacteria, archaeal mock communities are also commercially available, and are used as controls in sequencing microbial studies [53].

### 2.3. Negative Controls

Negative controls are typically blank samples that have been process alongside the samples in order to quantify and identify the possible contaminants introduced by the experimental method. Field blanks should also be included when natural environments are sampled (human gut, air, water, soil, etc.). As NGS is particularly likely to be affected by the presence of contaminants, the use of negative controls in such studies is mandatory [54]. Multiples negative controls can also be incorporate in a study design to assess to incorporation of contaminants at different step of the procedure [55,56].

There is currently no consensus on how to bioinformatically manage the negative controls. The OTUs identified in them are usually completely removed from the entire dataset [55]. However, such strategy could also take out OTUs that are naturally present in the samples and reduce the observed diversity. More sophisticated techniques, such as the use of quantitative polymerase chain reaction (qPCR) data to correct absolute counts [57], have also been developed, but these are not broadly accepted as they can also skew the results. Furthermore, even if no corrections are applied to the samples according the OTUs found in the negative controls, they can act as a good indication of contamination and help construct a certain level of trust over the conclusions of a study using NGS.

In short, the use of NGS in microbial ecology can be a double-sided sword, as its power of analysis makes it more vulnerable to contaminations and technical biases. Precautions in the form of positives and negatives controls must be taken to ensure the validity of the results it produces and the conclusions it can lead to.

### 2.4. Data Transformation

Common outputs of sequence data processing pipelines include OTU and taxonomic tables that contain the identification number, the abundance (absolute counts) and taxonomic information of the OTUs in each sample. In order to compare the samples truthfully with one another, mathematical transformations must be applied to theses tables. They account for the sequencing depth and allow diversity comparisons, both for alpha and beta diversity.

## 3. Sequencing Depth

Sequencing depth can be defined as the number of reads obtained in a sample. It depends on the NGS platform used and the higher the sequencing depth, the more likely it is that diversity coverage will be attained [58]. Sequencing depth can affect diversity measures, as samples with more reads may appear richer and cluster differently in multivariate analyses. In order to counterbalance this effect, it is essential to normalize the data, so that all samples are brought down to the same sequencing depth or so they are compared on a relative basis. It is always recommended to try different data normalizing methods because the mentioned biases can remain present and can sometimes be considerable. One way to verify this trend is to add information about the number of reads per sample into the metadata before normalizing and see if samples with higher numbers of reads tend to cluster together.

Data normalization methods can include rarefaction or normalization. Rarefaction creates a subsampled data set by randomly sampling the input sequences up to a giving number. Samples with fewer sequences than the requested rarefaction depth are not included in the analyses. The outputs are diversity curves based on the number of sequences in a sample; rarefaction curves. These types of curves provide insightful information about how much microbial diversity is covered. If plateaus of richness and diversity are attained after a certain number of sequences per sample, they signify that sequencing efforts were sufficient enough to cover all of the diversity in the sample. Different rarefaction depth values should be tested. Two important considerations are: (1) finding the highest value for which the majority of samples would be included, and (2) finding the highest value that provides the best coverage plateau. The Vegan package using the R program can be used to rarefying the samples: https://rdrr.io/rforge/vegan/man/rarefy.html.

As an alternative to rarefaction, normalization accounts for uneven sample sequencing depth and attempts to correct compositionality. In other words, samples represent a fraction of the ecosystem and the observed sequences are relative abundances; therefore, the data are compositional. In general, normalization procedures attempt to minimize the technical variability between samples and sample-specific dispersion [59]. A novel normalization technique, CSS (cumulative sum scaling) by metagenomeSeq, corrects the bias associated with the assessment of differential abundance to a pre-determined percentile by dividing raw counts by the cumulative sum of counts [60]. It is not recommended to use normalized data with presence/absence metrics like binary metrics or unweighted UniFrac, because CSS methods are abundance-based. Although used mainly for differential abundance analysis (statistically significant differences in microbe abundance across samples), DESeq can also be used as another data normalization alternative to rarefaction [59,61,62]. The Differential Abundance section of this paper addresses the DeSeq method in the context of differential abundance analysis.

Normalization and rarefaction present both advantages and disadvantages. When a subsample is generated to an even depth (rarified), some observations are discarded which reduces the ability to detect differences in diversity measures [63]. Although there is a definite reduction in resolution, the simplicity and clarity of the method can be worth the loss of a few reads. Furthermore, microbial communities are often different enough that the loss of a few reads won’t affect the overall measure of diversity [62]. Despite normalizing data using CSS being a promising technique, it should be used with caution as it can dramatically exaggerate the low-abundance taxa which can lead to their over-representation in a CSS normalized data set [63]. Also, DESeq produces negative values for Operational Taxonomic Units (OTUs) with low abundances as a result of its log transformation. Some diversity metrics, like Bray-Curtis, cannot be used with negative values and therefore can’t be used to analyze a data set normalized by DESeq. The key is to verify the results using multiple normalizing approaches, as different methods can complement each other depending on the goal of the research. Verifying the normalization outcome include considering the bias introduced by the method and stating it as a limitation. The latter limitation could be compensated by a second method, which corrects the bias. For example, the CSS normalization corrected the bias in the assessment of differential abundance introduced by total-sum normalization (TSS). It is important to consider that normalization is a highly debated topic and there is currently no consensus from experts on which normalization method is better [64].

### Alpha and Beta Diversity

The measurement of species diversity was first introduced by Whittaker and defined as the number of species and their proportion within one sampling site [65]. There are different ways to measure alpha diversity depending on the context of the study. A list of indexes is presented by Magurran and McGill [66]. The number of observed OTUs, Chao1, Shannon and Simpson are commonly used alpha diversity measures and have been shown to perform well in the context of bioaerosol exposure studies [26,27,67]. More specifically, Chao1 is a richness estimator. The higher the number of unique OTUs in a sample, the higher the value of the Chao1 index. For Shannon and Simpson, the species richness is combined with the abundance to give one diversity measure. The Simpson index represents the probability of two randomly selected OTUs from the same sample, being of/from the same species. The output values are bounded between 0 and 1, where 0 represents the highest diversity. Shannon output values start at 0, and higher values are associated with higher diversity.

An important factor to consider when choosing an alpha diversity measure for comparing sets of samples is the gene marker used for HTS, as the use of some markers may limit your choices of indexes. For example, PD Whole Tree is a phylogenetic alpha diversity measure and is defined as the minimum length of all phylogenetic branches acquired to span a given set of taxa on the phylogenetic tree [68]. Thus, the use of a reliable phylogenetic tree is necessary when applying the PD Whole Tree analysis. Compared to the markers for 16S bacterial and archaea genes, the fungal ITS gene marker is subject to intraspecific variability [69]. The construction of a phylogenetic tree is not recommended due to the possibility of obtaining different results using the same dataset but with different tree construction methods (data not shown). Every metric has different strengths and limitations. Technical information on each metric is available in ecology textbooks and is beyond the scope of this paper.

As alpha diversity was a measure of diversity inside individual samples, beta diversity compares the microbial composition between samples from different environments [70]. It measures the differences in overall microbial profiles. The output of beta diversity measures is a distance matrix containing a dissimilarity value for each pairwise comparison (each sample compared to another). Before any comparison can be accurately made, samples must be normalized as described above, normalized by relative abundance inside each sample, or rarefied so that they all have the same sequencing depth [59,60]. There are a number of metrics for beta diversity measurements that can be classified into two categories: those that use phylogenetic information (rely on the quality of the constructed phylogenetic tree) and those that do not, which are formally known as non-phylogenetic methods [71,72,73,74]. One of the most used phylogenetic beta diversity measures is Unique Fraction (UniFrac), which measures the degree of unique evolution of one microbial community compared to others [75]. With the assumption that closely related species have similar genetic functions, the abundances of phylogenetically similar taxa have less importance when using UniFrac for beta diversity measurements [76]. Quantitative measures (e.g., weighted UniFrac) are suited for revealing community differences that are due to changes in relative taxon abundance (e.g., when a particular set of taxa is more abundant in bioaerosol samples compared to the source of aerosolization). Qualitative measures (e.g., unweighted UniFrac) consider the presence/absence of OTUs and are most informative when bioaerosol microbial communities differ according to various factors such as temperature, relative humidity, season, and time. In fact, information on relative abundance can sometimes mask significant patterns of variation in which taxa are present [71]. The Bray-Curtis Dissimilarity Index is one of the most popular non-phylogenetic measures [77]. It quantifies the compositional dissimilarity between two different samples, based on the counts from each sample. The Bray–Curtis dissimilarity is bounded between 0 and 1, where 0 means the two samples have the same composition and 1 means the two samples do not share any taxa. It is not considered a distance because it does not satisfy the triangle inequality rule and should be called a dissimilarity to avoid confusion. Bray–Curtis and Jaccard indices both use rank-order but the Jaccard index is metric while Bray-Curtis is semi-metric.

Alpha and beta diversity indexes can be calculated using the scripts described in QIIME1 at http://qiime.org/scripts/alpha_diversity.html and http://qiime.org/scripts/beta_diversity.html or using QIIME2 at https://forum.qiime2.org/t/alpha-and-beta-diversity-explanations-and-commands/2282. Alternatively, the Vegan package can also be used for more control over options and parameters: https://cran.r-project.org/web/packages/vegan/vegan.pdf.

## 4. Visualization Tools

### 4.1. Alpha and Beta Diversity

Once distances/dissimilarities between samples are computed, hierarchical clustering can be used to detect patterns of sample grouping. Samples with similar microbial compositions are grouped together in the branches of a dendrogram [78]. Hierarchical clustering is a useful tool for sample grouping visualization but should be coupled with additional statistical tests [32]. Moreover, the information in the distance matrices generated can be displayed in a dimensional space (two or three orthogonal axes) for better visualization of the sample closeness. Two popular ordination techniques in microbial ecology are non-metric multidimensional scaling (NMDS) and metric multidimensional scaling (MDS). The classic example of multidimensional scaling is the Principal Coordinates Analyses (PCoA) [32,75,79]. MDS algorithms aim to place each sample in *N*-dimensional space such that the inter-sample distances are preserved as much as possible. Each sample is assigned coordinates in each of the *N* dimensions. The number of dimensions on an MDS plot can exceed 2 and is specified a priori. Choosing *N* = 2 optimizes the object locations for a two-dimensional scatterplot. The stress value associated with the MDS expresses the goodness of fit of the ordination and is better when nearing zero. The accuracy of the PCoA plot can be evaluated using jackknifing which is an iterative resampling procedure where one OTU from the data set is omitted in each iteration. Then, the average is represented on a PCoA plot with variance represented as confidence ellipsoids [75]. On the contrary, the position of samples in NMDS represents the rank order of inter-sample distances. In general, both ordination techniques should lead to similar conclusions and it is recommended to test both methods on each data set. To choose the method that is most appropriate for the dataset, there are several papers that are dedicated to the subject and that go into greater details [80,81,82]. Constrained ordinations differ from unconstrained ordinations, such as PCoA and MDS/NMDS, because they maximize the plot to display the greatest separation of samples from selected variables. On the other hand, unconstrained ordinations try to explain the variability of the dataset on a limited number of axis for every variable (dependent or independent), which can lead to less separation in clusters and a harder to detect trends [80]. Multiple versions of constrained ordinations are available, such as Canonical Analysis of Principal coordinates (CAP) [83] and Distance-Based Redundancy Analysis (db-RDA) [84].

It is advised to use both a robust unconstrained ordination (e.g., MDS) and constrained ordination (e.g., CAP), combined with appropriate statistical tests, to get the best picture out of a dataset [83].

### 4.2. Additional Visualization Tools

**Figure 2:**
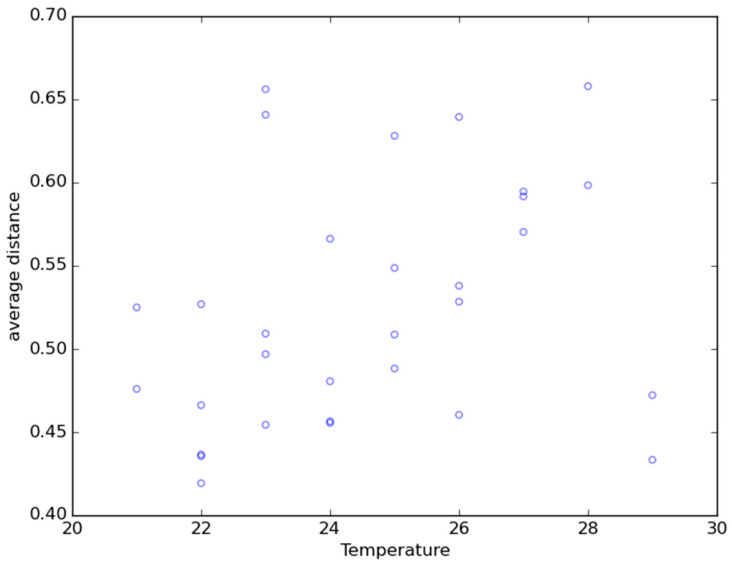
Scatterplot representing average distances between samples. The distances were calculated between groups of air samples collected in different wastewater treatment plants during summer and winter.

Creating a scatterplot representing average distances between samples (distance matrices), broken down by specified parameters (categories) is an alternative way to compare the microbial compositions of samples. The inputs are a distance matrix and a mapping file. The x-axis represents a category and must be numerical. In the primary state, each sample within the category will be compared to the other samples (or the one representing the secondary state) and an average of their distances will be calculated. The average distances will be plotted against a numerical category and are represented in the y-axis. The numerical category in the x-axis should preferably be linear and correlated somehow to the primary state. The points on the plot can then be colored according to another defined category. Thus, we have average distances between the groups we are comparing according to a linear parameter (e.g., variation of the microbial composition of bioaerosols according to days, temperature, etc.). An example of a scatterplot representing average distances between samples is presented in Figure 2. The distances were calculated between air samples collected in different wastewater treatment plants during summer and winter. The temperature did not affect the distance between air samples.

Similar to scatterplots, boxplots can be used to compare distances between categories of samples. The boxplots can compare distances within all samples of a category, as well as between different categories. Thus, individual-, within- and between-distances can be plotted. The input for a scatterplot is a distance matrix with the mapping file explaining the categories of samples. Statistical test comparing all combinations of paired boxplots can help determine which microbial distributions are significantly different from the others.

In addition to using NMDS and MDS plots, building a neighbor joining tree or a Unweighted Pair Group Method with Arithmetic mean (UPGMA) tree that compares samples, using a distance matrix as input, is another way to examine sample grouping. Neighbor joining is an agglomerative clustering method for creating phylogenetic trees. Typically used for trees based on DNA data, the algorithm requires knowledge of the distance between each pair of taxa. In this case, it is used to cluster samples. Compared to UPGMA, the advantage of neighbor joining is that it does not assume that all lineages evolve at the same rate [85].

Information in an OTU table can be visualized as a heatmap where each row corresponds to an OTU and each column corresponds to a sample. The higher the relative abundance of an OTU in a sample, the more intense the color at the corresponding position on the heatmap. The OTUs can be clustered by UPGMA hierarchical clustering, and the samples are presented in the order in which they appear in the OTU table. This is useful for establishing a general overview of the samples that have equal abundance of OTUs and are clustered together. However, identification of specific OTUs is difficult to visualize when the number of OTUs from the OTU table is very high. Therefore, presenting the OTUs in bar graphs taxonomic analyses are preferred for OTU identification. The Vegan package offers functions to generate all the plots mentioned in this section: https://cran.r-project.org/web/packages/vegan/vegan.pdf.

## 5. Statistical Analysis

### 5.1. Parametric VS. Nonparametric Statistics

Nonparametric statistics are not based on parameterized families of probability distributions [86]. Some examples of the typically used parameters are mean, median, mode, variance, range, and standard deviation. Unlike parametric statistics, nonparametric statistics make no assumptions about the probability distributions of the variables being assessed. The difference between parametric and nonparametric models is that the former has a pre-established number of parameters, while the latter determines the number of parameters depending on the dataset. In other words, the parameters are determined by the dataset in nonparametric statistics, and by the model in parametric statistics.

Since ecological datasets rarely conform to the normal distribution [87], parametric tests are often not the right fit. In order to use parametric tests on these datasets, one should verify that their characteristics are in line with the assumptions of the tests. The combined use of visual approaches (frequency distribution) and of a statistical test for normality, such as the Shapiro-Wilk test, is advised to confirm the normality of the dataset [88]. Sample size and dispersion (data spread in all groups) should also be checked before using a parametric test with data that do not have a normal distribution in order to choose the right test. For example, the 2-sample *t*-test and One-Way ANOVA assume equal variances and these options should not be selected when the dispersion of data in each group of samples is different. Usually, parametric tests have equivalent nonparametric tests that can be used as alternatives. Here are a few examples of related pairs of tests: 1-sample *t*-test and Wilcoxon; 2-sample *t*-test and Mann-Whitney test; One-Way ANOVA and Kruskal-Wallis. Even though parametric tests have more statistical power for detecting significance, nonparametric tests can be more suitable when a dataset is better represented by the median rather than the mean [89]. Also, nonparametric tests perform better with ordinal and ranked data compared to parametric tests that can only assess continuous data. Thus, nonparametric tests can better handle exceptions that cannot be removed [90].

According to the central limit theorem, if the mean accurately represents the center of the distribution of the dataset and the sample size is large enough (>30), one might consider a parametric test even with a non-normal distribution [88]. However, if the median is a better representative of the center of the distribution of the dataset, nonparametric tests can give more accurate results even with a large number of samples. It should be noted that when the sample size is very small, nonparametric tests are the only option. Overall, checking the assumptions associated with the statistical test is crucial for making the best choice as each one has its own data requirements [91].

### 5.2. Comparisons Using Alpha and Beta Diversity Measures

Alpha diversity index values obtained for each sample can be compared based on parametric or nonparametric tests that use multiple groupings of sample data. For example, air samples may be labeled as one of three types: outdoor control, sampling site 1 or sampling site 2. Statistics comparing each combination of two sample groups (outdoor control and sampling site 1; outdoor control and sampling site 2; sampling site 1 and sampling site 2) can be used. The results include the means and standard deviations of the alpha diversities of the two groups, along with the *p*-value of the statistical test. Based on these results, one can determine which groups of samples are significantly richer and more diverse than the others. Commonly used tests include paired or unpaired *t*-test and Wilcoxon test and the Kruskal-Wallis test.

### 5.3. Statistical Significance of Sample Groupings

The analysis of the strength and statistical significance of sample groupings using a distance matrix as the primary input can be used in combination with the previously discussed NMDS or MDS (PCoA) to further validate that the detected patterns of sample groupings are statistically robust. There are several methods available for analyzing the statistical significance of sample groupings using distance matrices. The suitability of these methods should be evaluated based on parametric or nonparametric features and on distance matrices that are constructed with metric, semi-metric or non-metric dissimilarities. The following tests are among the most used in microbial ecology studies, and are well suited for bioaerosol studies more specifically: Adonis ANOSIM, BIO-ENV, Moran’s I, MRPP, PERMANOVA, PERMDISP, and db-RDA (vegan package, R).

The Adonis test partitions distance matrices among sources of variation in order to describe the strength and significance that a categorical or continuous variable has in determining variation of distances. This is a nonparametric method and is almost equivalent to db-RDA, except when distance matrices are constructed with semi-metric or non-metric dissimilarities, which may result in negative eigenvalues. Adonis is very similar to PERMANOVA, though it is more robust because it accepts both categorical and continuous variables in the metadata mapping file, while PERMANOVA only accepts categorical variables [92]. Moreover, PERMANOVA is based on the ANOVA experimental design, but because it is a non-parametric test it analyzes the variance and determines the level of significance using permutations [93]. While ANOVA/MANOVA assumes normal distributions and Euclidean distance, PERMANOVA can be used with any distance measure as long as it is appropriate to the dataset. PERMDISP is a method that analyzes the multivariate homogeneity of group dispersion (variances). It determines whether the variances of groups of samples are significantly different. The results of both parametric and nonparametric significance tests are provided in the output. This method is generally used in combination with PERMANOVA [94]. MRPP is another method that tests whether two or more groups of samples are significantly different based on a categorical variable found in the metadata mapping file. Since MRPP is nonparametric, significance is determined through permutations [95]. ANOSIM tests whether two or more groups of samples are significantly different based on a categorical variable found in the metadata mapping file. Since ANOSIM is nonparametric, significance is also determined through permutations [96]. Similar to Adonis, db-RDA differs if certain non-Euclidean semi or non-metrics are used to produce the distance matrix, and negative eigenvalues are encountered. This difference will be apparent in the *p*-values, not the R^2^ values. BIO-ENV (BEST) finds subsets of variables whose Euclidean distances are maximally rank-correlated with the distance matrix. For example, the distance matrix might contain UniFrac distances between communities, and the variables might be numeric environmental variables (e.g., pH and latitude). Correlations between the community distance matrix and Euclidean environmental distance matrix is computed using Spearman’s rank correlation coefficient (rho). This method will only accept continuous or discrete numerical categories [97,98,99]. Interestingly, this method accepts more than one category to explain variation between groups of samples. Moran’s I is another method that uses numerical data to identify which type of numerical variables explains sample grouping [100]. In short, a multitude of tests have been developed to statistically test the significance of grouping. One should ensure that the selected method is appropriate for the type of data being analyzed and for scientific questions it is trying to answer. Table 1 presents a summary of the applicable methods with the important parameters to consider when choosing one.

### 5.4. Correlations

One common application of distance matrix comparison techniques is to determine if a correlation exists between an ecological distance matrix (e.g., UniFrac distance matrix) and a second matrix derived from an environmental parameter that is numeric/continuous (e.g., differences in pH, temperature, or geographical location). For example, one might be interested in knowing if aerosol samples with different pH levels are more different from one another than from aerosol samples with similar pH levels. If so, this would indicate a positive correlation between the two distance matrices. Mantel correlation tests allow for the comparison of two or more distance/dissimilarity matrices to determine if there is a correlation. It tests the hypothesis that distances between samples within a given matrix are linearly independent of the distances within those same samples in a separate matrix. 

**Figure 3:**
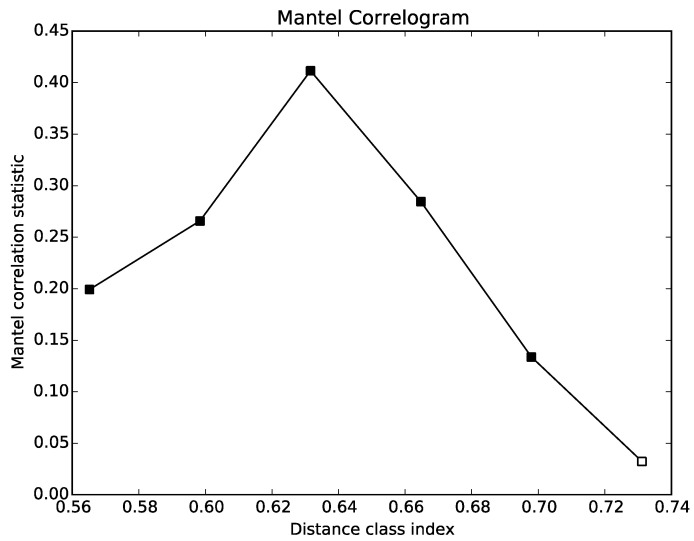
Correlation of two distance matrices (weighted and unweighted unifrac) on air samples from wastewater treatment plants by the Mantel correlogram matrix correlation test. A filled-in point on the plot indicates that the Mantel statistic was statistically significant.

A Mantel correlogram produces a plot of distance classes versus Mantel statistics. Briefly, an ecological distance matrix and a second distance matrix (e.g., spatial distances, pH distances, etc.) are provided. In the second distance matrix distances are split into a number of distance classes (this number is determined by Sturge’s rule). A Mantel test is applied to these distance classes versus the ecological distance matrix. The Mantel statistics obtained from each of these tests can then be plotted in a correlogram. A filled symbol on the plot indicates that the Mantel statistic was statistically significant [101]. An example of a mantel correlogram plot is presented in Figure 3, using air samples from wastewater treatment plants compared with weighted and unweighted distance matrices.

Moreover, correlations between abundances (relative or absolute) and numerical metadata can also be used to correlate features to sample metadata values. Several methods are available to accomplish this. Pearson is a parametric and linear measure of correlation. It is a scaled measure of the degree to which two sequences of numbers co-vary. For correlated sequences, Pearson > 0, and for anticorrelated sequences, Pearson < 0 (uncorrelated implies Pearson = 0). The Spearman correlation is a nonparametric measure of the correlation between two sequences of numbers. Kendall’s Tau is an alternative method of calculating correlations between two sequences of numbers. However, it is slower and utilized less often than Spearman or Pearson scores [102]. Statistics can be added to these correlation approaches in order to generate *p*-values to confirm the correlation scores obtained. Bootstrapping is the most robust procedure for calculating the *p*-value of a given correlation score. Bootstrapping takes the input sequences, randomly changes the order of one, and then recomputes the correlation score. The *p*-value represents the number of times (out of the given number of permutations) that the score of the permuted sequence pair was more extreme than the observed pair. Bootstrapping is preferred when information about statistical distributions is unknown (https://cran.r-project.org/web/packages/bootstrap/bootstrap.pdf).

Finally, the correlation between samples in terms of their taxonomic composition can also be computed. This is useful for determining if the taxonomic compositions of mock communities that were assigned using different taxonomy assigners are correlated. Another usage is to compare the taxonomic compositions of several mock community samples to a single known sample community. In general, correlations in the taxonomic composition between different groups of samples can be useful (e.g., aerosol samples collected from different sites). The correlation coefficient, an associated confidence interval, and *p*-values (nonparametric or parametric) should also be included using the method discussed previously.

## 6. Taxonomic Analyses

The taxonomic analysis uses an OTU table containing taxonomic information as input data. This information was obtained by comparing the consensus nucleotide sequence of the OTU to a public database. The databases should be chosen based on the gene marker used for the study. Greengenes is a 16S rRNA gene database suited for bacterial diversity [103]. UNITE is more appropriate for the fungal ITS gene [104]. SILVA is a wider database of small (16S/18S, SSU) and large subunit (23S/28S, LSU) rRNA sequences for all three domains of life (Bacteria, Archaea and Eukarya) [105]. SILVA is the most up-to-date database and should be chosen over other databases as they tend to be outdated. Even though, some might go to the species rank, these tend to be unreliable. Next, the taxonomic level for which the summary information is provided is designated. This level will depend on the format of the taxon strings that are returned from the taxonomy assignment step. The taxonomy strings that are most useful are those that standardize the taxonomic level with the depth in the taxonomic strings. For instance, for the RDP classifier taxonomy: level 2 = Domain (e.g., Bacteria), 3 = Phylum (e.g., Firmicutes), 4 = Class (e.g., Clostridia), 5 = Order (e.g., Clostridiales), 6 = Family (e.g., Clostridiaceae), and 7 = Genus (e.g., Clostridium). Although, the relative abundance of each taxonomic group is the most used technique to compare taxa, raw counts can also be used for an absolute abundance. Results can be displayed with bar or area charts comparing taxonomy between groups of samples or between all individual samples. In addition, each pair of samples can be compared and the number of their shared OTUs is displayed in order to focus only on common OTUs between groups of samples.

Furthermore, the inclusion of taxonomic information in the mapping file allows NMDS or MDS plots to be colored based on taxonomy. More specifically, results displayed on principal coordinate plots can be colored based on any of the metadata fields in the mapping file. Coloration of the plots based on the relative abundances of each taxon can help in distinguishing which taxonomic groups are responsible for the sample grouping patterns.

Taxonomic analyses can also include the calculation of the ratio of abundance of specified taxonomic groups. This method is based on the microbial dysbiosis index described by Gevers and his coauthors [106]. Microbial Dysbiosis index (MD-index) is used as an indicator of the microbial imbalance within samples. One should specify the taxonomic groups to be used for the analyses according to their susceptibility to being affected by the different environmental conditions that define the samples. This index provides the option to choose the numerator and the denominator of the log ratio. The index must include the taxonomic groups that will be tested for increase (numerator) and decrease (denominator). For example, the ratio comparing firmicutes and proteobacteria would have firmicutes as the numerator and the proteobacteria as the denominator. To determine the taxonomic biomarkers, one can use a distance matrix plotted on ordination and validate which variable in the metadata mapping file best/most explains the variation observed, and then use taxonomic analyses to visualize the taxonomic composition of the samples based on the variable chosen. That way, it is possible to determine which taxonomic groups exhibit differential abundance and can be used for the specified MD-index. The comparisons between samples based on microbial dysbiosis and the categories they belong to in the metadata mapping file can help determine which environmental condition creates a microbial dysbiosis. In bioaerosol studies, the analyses of dysbiosis can be very useful in determining if there is a microbial imbalance between a given source and the aerosols released.

Finally, identification of the core microbiome is another example of taxonomic analyses that provide useful information on the ecology of bioaerosols. The core of a microbiome is defined as the minimum community of microbes that is essential for a well-functioning ecosystem. This concept that has mostly been applied to the gut ecosystem may also be applicable to bioaerosols [107,108]. The identification of the species that are found in a certain percentage (e.g., 50% to 95%) of all aerosol samples from a specific environment can determine the core microbial composition (core microbiome) of the environment being investigated. The importance of characterizing a core microbiome for each environment is extremely evident when searching for biomarkers of bioaerosol exposure in hazardous environments. The characterization of these biomarkers plays a key role for better evaluating the risk of bioaerosol exposure and will help in the standardization of bioaerosol studies.

## 7. Differential Abundance

Differential abundance analyses allow for the identification of OTUs that are differentially abundant across two sample categories in the mapping file (e.g., outdoor and indoor air samples). Two parametric tests are available for such analyses: MetagenomeSeq zero-inflated Gaussian (ZIG) and DESeq2 negative binomial Wald test. It is recommended to have at least five samples in each category to apply these methods. However, caution is required as parametric tests assume a normal distribution and perform poorly when assumptions about the data are not met. The input is a raw (not normalized, not rarefied) matrix with uneven column sums. With these techniques, it is still recommended to remove low depth samples (e.g., below 1000 sequences per sample), and low abundance/rare OTUs from the datasets. It is also possible to remove low variance OTUs across the entire dataset to limit the number of comparisons being made and lower the statistical corrections being applied to the resulting *p*-values. QIIME offers a diagnostic plot along with the differential abundance analyses. The DESeq2 method should not be used if the fit line on the dispersion plot is not smooth, if there are big gaps in the point spacing, or if the fitted line does not look appropriate to the data [32]. DESeq2 is stronger when used with very small datasets, while MetagenomeSeq’s fitZIG uses an algorithm better suited for larger sized libraries with over 50 samples per category (the more the better). The results are presented in the form of a list of all of the OTUs in the input matrix, along with their associated statistics and the *p*-values that determine the statistical power of the differential abundance in the compared categories. These methods can be used in combination with the rarefied approaches to compare their outcomes. This manuscript is meant as a guide presenting recommended analyses for use in bioaerosol microbial ecology studies and the tools to achieve them. However, more detailed technical information can be found in the original papers describing the methods [64,67,109].

In the context of differential abundance analyses, here defined as rarefied approaches are statistical tests that compare OTU frequencies in sample groups and ascertain whether or not there are statistically significant differences between the OTU abundances of different sample groups. Rarefying the samples prevents zero-variance errors and spurious significance for low abundance OTUs and focuses on the abundant OTUs, which likely play the most important role in the differential abundance. Put differently, the most abundant OTUs are the ones of interest in differential abundance analyses. Thus, losing low abundance OTUs is worth it. Examples of statistical test that can be applied to rarefied data are the G-test, Kruskal-Wallis, ANOVA, Mann-Whitney U and *t*-test. Each test has its own null and alternate hypotheses and its own assumptions. It is important to check the sample size requirements, assumptions, and the null and alternate hypotheses of each test in order to determine which is most appropriate for the dataset. Documentation on QIIME and R packages provides useful information on the subject, as does key literature on the subject of statistics in ecology [110]. The three nonparametric tests (Kurskal-Wallis, Wilcoxon, and Mann-Whitney U) are most suited for bioaerosol sequencing data when the statistical distribution is not known. The *t*-test and Mann-Whitney U test may only be used when there are two sample groups, while Kruskal-Wallis can also be used when three or more groups of samples are compared (e.g., outdoor, indoor, source and samples).

A new method emerged that produces exact sequence variants (ESVs) instead of OTUs for a greater resolution than OTU-based methods. DADA2 processes data from fastq files, removes errors and chimeras, and produces sample abundances and taxonomic assignments [44]. Other synonyms of ESVs are amplicon sequence variant (ASV), zero radius OTU (ZOTU), or simply an OTU defined by 100% sequence similarity. ASVs prone a better amplicon resolution by distinguishing sequence variants differing by one nucleotide. ASVs most prominent advantage is the combination of the benefits from overcoming limitations inherent to closed-reference and de novo methods. For instance, closed-reference OTUs cannot document biological variations outside of the reference database used for their construction. On the other hand, the validity of de novo OTUs outside of the dataset in which they were defined is also questionable, which make cross-studies comparison invalid. While ASVs capture all biological variations present in a dataset, and ASVs inferred from a given dataset can be reproduced in future datasets and validly compared [111]. However, ASVs method also comes with its share of limitations. Allowing 100% sequence similarity may lead to a wrong differentiation between the SNPs of the same species. In addition, the zero percent difference may give an extremely high number of ASVs in a sample, which, in return, causes the missing of the core microbiome information’s (unpublished data). Above all, the same genome can contain multiple ASVs if there are multiple copies of the targeted gene. For this matter, ASVs can be validly compared between studies, only when the same primers were used on the targeted gene. Furthermore, the high variability of the ITS region makes us reconsider the automatic replacement of the traditional OTUs by ASVs. To sum up, ASVs and de novo OTUs are more precise in describing diverse biological sequences in a less represented environment in reference databases like bioaerosols, compared to closed-reference OTUs. Most importantly, no matter the methodology used, downstream analyses should consider the methodological differences, accordingly.

## 8. Conclusions

The analysis of microbial diversity is becoming a crucial component in several fields of scientific research, and bioaerosols is no exception. Many of the bioinformatics tools used to study microbial diversity were developed for researchers comfortable with a command line environment. This manuscript is intended as a guide to the types of useful bioinformatics tools that provide a thorough investigation of the microbial communities of bioaerosols. Many questions can be answered, hypotheses confirmed and critical thinking can be triggered by such analyses. Thus, the main goal is not to provide command lines about how to perform the analyses, but to offer important information and insight on tests typically used in microbial ecology. We do this by providing examples of their application in bioaerosols studies. Bioinformatics tools are still underutilized by bioaerosol scientists and they can, in some cases, lead to spurious analyses and interpretations. The authors hope that this work represents a popularization of bioinformatics in the study of bioaerosols and will provide a good source for the «dos and don’ts» when conducting a critical microbial community study.

## Figures and Tables

**Table 1:** Summary of methods to test the significance of sample grouping.

Methods	Type of Statistics	Type of Variables	Comment
Adonis	Nonparametric	Categorical and Numerical	Semi-metric and non-metric dissimilarities
ANOSIM	Nonparametric	Categorical	-
BIO-ENV	N/A	Numerical (continuous or discrete)	Rank-correlation between Euclidean distances and distance matrix
Moran’s I	N/A	Numerical	Identify spatial configuration in samples
MRPP	Nonparametric	Categorical	-
PERMANOVA	Nonparametric	Categorical	Uses an ANOVA experimental design and returns pseudo-F and a *p*-value
PERMDISP	Parametric and nonparametric	Categorical	Analysis of multivariate homogeneity of variances
db-RDA	Nonparametric	Categorical	A category in the metadata can be specified to explain the variability between samples
